# Life History Traits and Niche Instability Impact Accuracy and Temporal Transferability for Historically Calibrated Distribution Models of North American Birds

**DOI:** 10.1371/journal.pone.0151024

**Published:** 2016-03-09

**Authors:** Guinevere O. U. Wogan

**Affiliations:** 1 National Audubon Society, Emeryville, CA, United States of America; 2 Department of Environmental Science, Policy, and Management, University of California, Berkeley, CA, United States of America; 3 Museum of Vertebrate Zoology, University of California, Berkeley, CA, United States of America; University of Colorado, UNITED STATES

## Abstract

A primary assumption of environmental niche models (ENMs) is that models are both accurate and transferable across geography or time; however, recent work has shown that models may be accurate but not highly transferable. While some of this is due to modeling technique, individual species ecologies may also underlie this phenomenon. Life history traits certainly influence the accuracy of predictive ENMs, but their impact on model transferability is less understood. This study investigated how life history traits influence the predictive accuracy and transferability of ENMs using historically calibrated models for birds. In this study I used historical occurrence and climate data (1950-1990s) to build models for a sample of birds, and then projected them forward to the ‘future’ (1960-1990s). The models were then validated against models generated from occurrence data at that ‘future’ time. Internal and external validation metrics, as well as metrics assessing transferability, and Generalized Linear Models were used to identify life history traits that were significant predictors of accuracy and transferability. This study found that the predictive ability of ENMs differs with regard to life history characteristics such as range, migration, and habitat, and that the rarity versus commonness of a species affects the predicted stability and overlap and hence the transferability of projected models. Projected ENMs with both high accuracy and transferability scores, still sometimes suffered from over- or under- predicted species ranges. Life history traits certainly influenced the accuracy of predictive ENMs for birds, but while aspects of geographic range impact model transferability, the mechanisms underlying this are less understood.

## Introduction

Environmental niche models (ENMs) estimate the relationship between species records at sites and the environmental and/or spatial characteristics of those sites, and extrapolate species distribution data in space and time based on a statistical model in order to represent the realized environmental niche of species [1–4]. The accuracy of ENMs can be affected by the life history characteristics of the organisms under study [5–9]. While many studies address how life history traits impact model accuracy for different taxonomic groups, there are many fewer studies that examine the influence of differing life histories on model transferability [6, 10, 11]. Model transferability refers to how well a model built using environmental parameters from one geographic location or time performs when projected into a different geographic location or time [12, 13]. Although ENMs generally perform well when predicting within the same environment or climate [14, 15], when they are projected across geographic or climatic spaces they can become less accurate and reliable [16], thus model transferability is an important consideration in studies that involve forecasting or hindcasting [17, 18].

Within taxonomic groups, individual species differences greatly impact ENM transferability, in fact, species differences have a greater affect on transferability than model choice for some organisms such as found among plants [6, 11], mammals [19] and butterflies [11]. Having a strong contextual framework for the role of differing life history characteristics can allow us to make predictions of how well models will perform when they are forecast to the future or hindcast to the past. This is particularly important to consider when forecasting ENMs for climate based conservation planning under projected climate change [20–22], or when hindcasting ENMs to generate phylogeographic and biogeographic hypothesis [23]. If we have a solid knowledge of the life history traits that impact transferability for a group of organisms, and an understanding of how those traits affect transferability, then we have a framework for model interpretation. For example, Dobrowski *et al*. [6] demonstrate that plant species with high dispersal capability are more easily modeled, projected, and interpreted because models for these organisms have high predictive accuracy and transferability. Furthermore, Kharouba *et al*. [10] found that models for butterfly species with narrower environmental niches were better predicted and projected than those with wider niches. This framework becomes critical when model validation is not possible, such as when forecasting to future predicted climate change.

Devising means of testing transferability is not always simple. Projecting across climates is complicated because model validation is much more difficult than when projecting within the same conditions for which the model was built [24]. With forecasting, model validation is confounded by the fact that true model validation cannot happen without the passage of time [24]. Therefore, an understanding of how well the models perform under analogous (but likely changing) climatic simulations is desirable to gain insight into model behavior. Under analogous conditions, all else being equal, one would expect very high model transferability, especially when considered over shorter timeframes. However, there are few studies that have examined this, and a recent study has identified this knowledge gap [25].

For birds, some of the life history traits that have been examined with regard to ENM performance include range size, conservation status, migratory behavior, rarity, endemism, body mass (size), habitat structure, and wetland affinity (reviewed in McPherson and Jetz [5]). Of these traits, range size, migratory behavior, and wetland affinity have detectable impacts on model performance for South African bird species. Narrow ranged species models performed better than those for common species, non-migrant species models were better than those for migrants, models for endemics outperformed models for non-endemics, non-wetland species models outperformed wetland species models [5]. However, the characteristics that contribute to a successful interpolative model (e.g. a model built and projected within the same climatic and geographic environment), may not be the same as those that make for a successful extrapolative model (e.g. a model built using one climatic and geographic environment and then projected to a different climatic or geographic environment) [6]. Therefore, transferability as it relates to life-history characteristics should ideally be evaluated explicitly.

Here I examine the relationship between life history characteristics and model accuracy and transferability for a subset of North American birds. I use historical occurrence records in combination with historical climate data to forecast to the current climate and to validate the models. Using historical data to assess model accuracy and behavior is a highly informative approach to model validation, and is the only direct method to assess temporal transferability [11, 15, 19, 24]. I quantify model accuracy, parameterization, and transferability, and then use generalized linear models to relate differences in these measures to differences in life history characteristics among species. This is done over a relatively short time period (50 years) in a region where climate is roughly analogous, thus the expectation is that transferability should be very high unless they are impacted by either artifacts of modeling or life history characteristics. This study focused on species with relatively steady ranges over the past fifty years rather than those with documented range shifts. By doing so it becomes more straightforward to disentangle artifacts of modeling from the effects of life history traits on predictive accuracy and transferability.

## Methods

### Species and Life History Characteristics

Included species encompass a range of distributions (e.g. widespread and common WC, widespread but rare WR, narrow endemic but common NEC, narrow endemic but rare NER), and life-history characteristics (Table 1). To gain additional insight into the range characteristics and habitat access for each species, I generated observed frequency maps using eBird [26]. The ebird database records observations and includes site records both with the known distribution as well as migrants, it provides a visual heuristic to assess which species might be impacted by dispersal limitations that prevent them from accessing suitable environments. The other life history characteristics include migratory status, habitat preferences, conservation status, population trend, and body size. I categorized the migratory status of each species as neotropical migrant for migration to tropical areas of Central or South America (N), temperate migrant for migration within temperate North America (T), or resident if they persist in the same area year round (R). Primary habitat preferences were categorized as scrub, woodland, grassland, wetland, desert, or shoreline habitats. Life histories were characterized with guidance from Gough et al. [27], the All about Birds website [28] and the Birds of North America Online [29], body mass data are from [30]. The IUCN Red List was used to assess conservation status and population trends [31], and species identified as one of the National Audubon Society’s 20 common birds in decline are indicated [32]. The two conservation traits (conservation status and population trend) are emergent characteristics that should capture dynamics of range change through time and have been used in other studies evaluating the role of species ecologies on ENMs [5]. Species with threatened conservation status for example are often threatened by limited range extent or small population size and may be as difficult to model as rare species [5]. On the other hand, species that are undergoing a population decline or expansion are potentially out of equilibrium and may present a different challenge for modeling [5, 18]. Since population trend data for two species (Field Sparrow and Eastern Meadowlark) conflict, separate analyses using both classification schemes were conducted.

**Table 1:** The species included in these analyses and their life history traits.

AOU	Common Name	Species Name	Range	Migratory Status	Habitat	Conservation & Population	Body Mass
AOU 10	Common Loon	*Gavia immer*	WC	T	E	LC-d	4
AOU 172	Northern Pintail	*Anas acuta*	WC	T	E	LC-d*	3
AOU 240	Broad-winged Hawk	*Buteo platypterus*	WC	N	W	LC-i	2
AOU 242	Swainson's Hawk	*Buteo swainsoni*	WC	N	G	LC	3
AOU 251	Golden Eagle	*Aquila chrysaetos*	WR	T	G	LC	4
AOU 271	Prairie Falcon	*Falco mexicanus*	WC	T	G	LC	3
AOU 295	Greater Sage Grouse	*Centrocercus urophasianus*	NER	R	S	NT-d	4
AOU 302	Greater Prairie Chicken	*Tympanuchus cupido*	NER	R	G	V-d	3
AOU 335	Clapper Rail	*Rallus longirostris*	WR	R	E	LC-d	2
AOU 360	Sandhill Crane	*Grus Canadensis*	WC	T	E	LC-i	4
AOU 376	Piping Plover	*Charadrius melodus*	NER	T	B	NT-i	1
AOU 379	Mountain Plover	*Charadrius montanus*	NER	N	G	NT-d	1
AOU 382	American Oystercatcher	*Haematopus palliates*	WR	R	E	LC	3
AOU 412	Long-billed Curlew	*Numenius americanus*	WC	N	G	LC-d	2
AOU 691	Burrowing Owl	*Athene cunicularia*	WR	N	G	LC-d	2
AOU 939	Lewis' woodpecker	*Melanerpe lewis*	WR	T	E	LC-d	2
AOU 971	Black backed woodpecker	*Picoides arcticus*	WR	R	W	LC	1
AOU 1203	Eastern Kingbird	*Tyrannus tyrannus*	WC	N	G	LC-d	1
AOU 1252	Loggerhead Shrike	*Lanius ludovicianus*	WC	T	W	LC-d*	1
AOU 1317	Yellow-billed Magpie	*Pica nuttalli*	NEC	R	W	LC	2
AOU 1341	Tree Swallow	*Tachycineta bicolor*	WC	T	E	LC	2
AOU 1361	Boreal Chickadee	*Poecile hudsonicus*	WR	R	W	LC-d*	1
AOU 1370	White-breasted Nuthatch	*Sitta carolinensis*	WC	R	W	LC-i	1
AOU 1372	Brown-headed Nuthatch	*Sitta pusilla*	NEC	R	W	LC	1
AOU 1425	Ruby-crowned Kinglet	*Regulus calendula*	WC	T	W	LC-i	1
AOU 1483	Wood thrush	*Hylocichla mustelina*	WR	N	W	LC-d	1
AOU 1575	Yellow Warbler	*Dendroica petechial*	WC	N	S	LC	1
AOU 1595	Palm Warbler	*Dendroica palmarum*	WC	N	S	LC-i	1
AOU 1804	Brewer's Sparrow	*Spizella breweri*	WC	N	S	LC-d	1
AOU 1805	Field Sparrow	*Spizella pusilla*	WC	T	S	LC-i*	1
AOU 1814	Grasshopper Sparrow	*Ammodramus savannarum*	WR	N	G	LC-d*	1
AOU 1837	Chestnut-collared Longspur	*Calcarius ornatus*	NER	T	G	NT-d	1
AOU 1880	Eastern Meadowlark	*Sturnella magna*	WC	T	G	LC-i*	1
AOU 1916	Baltimore Oriole	*Icterus galbula*	WC	N	W	LC	1
AOU 1931	Black Rosy-Finch	*Leucosticte atrata*	NER	T	O	LC	1
AOU 1958	Evening Grosbeak	*Coccothraustes vespertinus*	WC	T	W	LC-d*	1
AOU sms	Saltmarsh Sparrow	*Ammodramus caudacutus*	NER	T	E	V-d	1

Range Scenario categories are widespread common (WC), widespread rare (WR), narrow endemic common (NEC), and narrow endemic rare (NER). Migratory status categories are neotropical migrant (N), temperate migrant (T), or resident (R). Habitat categories are scrub [S], woodland [W], grassland [G], wetland [E], desert [D], shoreline [B]. Conservation refers to the IUCN Red List assessment for each species. Least concern (LC), vulnerable (V), near threatened (NT). We further identified decreasing (-d), increasing (-i) population trends using the IUCN assessment data, and demarcated those species identified by the National Audubon Society as one of the twenty common birds in decline with an *. Body mass was categorized as cat 1 ≤100g, cat 2 100-500g, cat 3 500-1000g, cat 4 ≥1000g.

### Occurrence Data

The models built here are based on survey data from the National Audubon Society’s Christmas Bird Count (CBC) [33]. The CBC is a yearly survey of bird species across North America. CBC data are gathered and contributed by citizen scientists that carry out a 15-mile diameter survey on a single day over a three-week period in December and January. Yearly surveys at some sites stretch back decades, and increased participation over the past several decades has led to the initiation of many new CBC survey circles. Abundance data for each observed species are then compiled for each circle surveyed. The CBC data capture the early winter ranges of resident and migrant species. The long temporal and wide-spatial coverage of CBC data present a unique foundation for investigating how North American birds have responded to historical and recent climate change. The CBC has expanded to include sites that fall outside of the continental United States, however most of these survey sites are very recent additions, thus there is a trade-off between spatial extent and temporal extent when using these data. Since the focus of this study is on temporal transferability, and in order to maximize the length of time that could be evaluated, sites outside of the continental United States were excluded.

The CBC data were compiled from 1950 to 2010 and abundance information was collapsed into a simple presence or absence for each circle surveyed in each year. Data were binned by decade for each individual species, and only presence data were used here. Modeling was limited to presence data for multiple reasons; first since presence data are available for most macro-organisms (e.g. vouchered museum samples) but absence data are largely lacking, using the presence only data allows these results to be more generally comparable to modeling efforts used for other taxa or datasets. Second, since absences can be either a true absence (e.g. the species is not present in the survey site), or a false absence (e.g., the species is not detected although it is present) either more complex modeling techniques or more complex survey techniques are required to adequately characterize the nature of the absences [34]. To correct for species mis-identifications, a conservative approach in which a 5% threshold was applied to each circle for each species, thus if a species was recorded less than 5% of the years at that survey site it was considered absent.

### Environmental Data

Studies that predict species distributions under future climate change are almost exclusively reliant on climatic data for forecasting, and although the paleontological record provides insight into other potential environmental parameters, most ENM-based hindcasting analyses are also limited to climatic data. Therefore, to make this study more broadly comparable, only climate data were included in these analyses. Fortunately, climate has demonstrated utility in predicting distributional changes [35]. Monthly climate data for the continental U.S. were obtained for each year from 1950 to 1999 from the Parameter Regression of Independent Slope Model (PRISM) climate group [36]. Decadal averages (using the mean) were then produced for mean annual temperature, maximum temperature, and minimal temperature, and mean annual precipitation, and then 19 bioclim variables were produced (S1 Table). Data were utilized at 2.5 arc minutes.

### Environmental Niche Models

In order to test the temporal transferability of ENMs, I build models using historical data from fifty years of field surveys (1950 to 1999) in combination with historical climate data and project them to the “future” (in this study “future” refers to 1960 to 1999). The projected models were validated through comparison with survey data from the time period to which they have been projected. Model validation using serial temporal sampling is becoming more common (for recent examples [19, 25]), providing an independent means for assessing model accuracy [24]. Since differences between modeling approaches are less impactful and insightful than other factors when evaluating transferability [6, 7, 11], rather than focusing on the transferability of different modeling techniques (see [11, 37] for recent reviews), I use a single model type with high extrapolative ability. Using historical data in this manner provides a more robust framework for evaluating temporal transferability than consensus modeling approaches since consensus approaches emphasis precision rather than accuracy [6].

**Fig 1:**
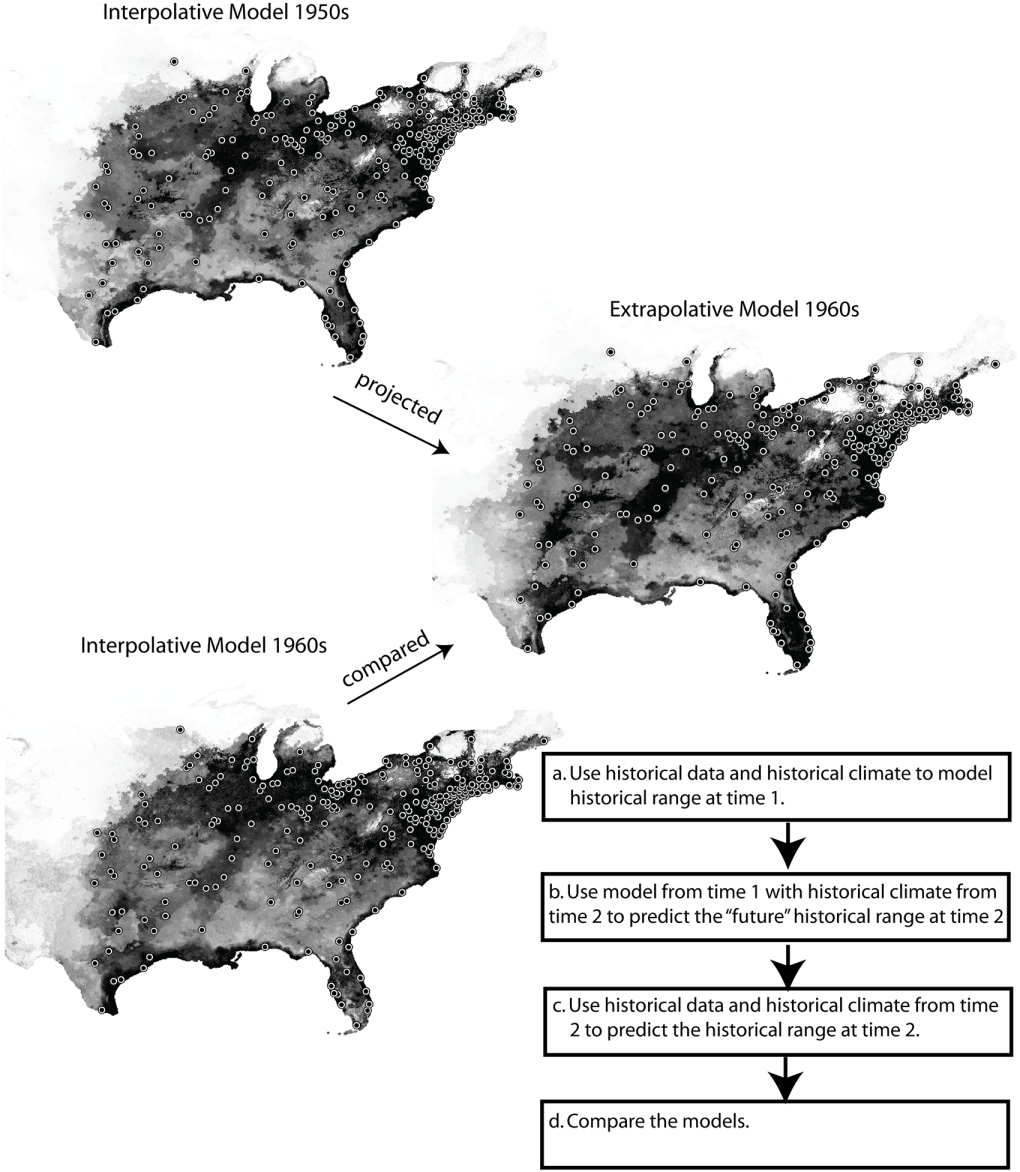
The modeling steps and validation procedure used to generate and compare the 555 ENMs that form the basis of analyses. Depicted are ENMs for the Eastern Meadowlark built for the 1950s and 1960s. The left column shows interpolative ENMs built using matched historical occurrence points and climate data, while the right column depicts extrapolative model that is built with data and climate from the 1950s and projected to the 1960s climate. Circles represent the occurrence points obtained from the CBC surveys during each decade.

Since there are recent studies investigating model variation with regards to geographic transferability [37] and temporal transferability [11] and both have found strong support for high transferability of MaxEnt models, I used MaxEnt 3.2e [38, 39] to build models and then project them into the “future”. ENMs using matched decadal occurrence records and climate data were generated for each decade, then projected forward in time, such that ENMs generated using 1950s occurrence points and 1950s climate, were projected to 1960s, 1970s, 1980s, and 1990s climates (Fig 1). Interpolative models (Im), are developed using occurrence data from a particular decade in conjunction with historical climate data from the same decade, and are expected to be good representations of the species’ distribution [14]. The extrapolative models (Em), which are the interpolative models projected onto a ‘future’ climate are more prone to error because they are built using climate data that differs from the climate to which they are projected [16].

A total of 555 models were constructed (15 models for each species). As previous analyses have demonstrated equivalent model performance with full versus subsets of environmental variables [16], and I had no *a priori* information by which to select variables across the modeled set of species [40], I used 19 bioclimatic variables for model building (S1 Table). However, there are two schools of thought regarding variable selection when using MaxEnt [41], one suggests including all reasonable predictor variables [38, 42, 43], the other suggests removing collinear variables [41]. In MaxEnt, allowing more predictor variables allows more complex model fitting, and by default MaxEnt determines which predictors to use based on the number of occurrences in the dataset [44]. MaxEnt uses weighting for variable selection, as well as an L1-regularization procedure that penalizes models in which predictor variables with little or no contribution are included [41, 45], thus it is generally thought to be less sensitive to model over-fitting than other methods [42, 43] (but see [46]). Furthermore, if the relationship among variables is not constant over time, the inclusion of multiple collinear predictors has been found to produce better performance and fit as compared to reduced variable models [47]. However, if variables are highly collinear, ecologically relevant predictors may be excluded if a collinear variable better explains the response variable [40, 48]. From a purely statistical standpoint, reducing collinear variables reduces the potential for mis-identification of critical variables [40]. To better understand the potential impact of collinearity among the predictor variables in the maxent models, and the constancy of variable relationships across decades, I calculated variance inflation factors (VIF) using the R package uSDM [49].

For each model a random starting seed was used, and up to 500 iterations were employed, 10000 pseudo absence points were generated from within the contiguous US, and duplicate presence points were removed such that each grid cell (~5km^2^) contained only a single point. This bias correction approach reduces spatial aggregation among presence points, although it does not correct for sampling gaps, it has been demonstrated to be an efficient and reliable means to correct for geographically biased sampling [50]. I used a conservative two-fold random cross validation approach (e.g. dividing the data into two groups) by randomly retaining 25% of the occurrence points for testing. The regularization value to minimize over-parameterization of the models was set to the MaxEnt default value based on findings from [42, 51] (but see [52] for further discussion).

AUC (area under the receiving operating characteristic curve) [53] is a metric commonly used to assess model performance in GIS modeling, since it is unbiased and threshold independent [54, 55]. The AUC is (or nearly is) prevalence independent [53, 56, 57], and instead depends on the probability of the model correctly ranking presence vs. absence sites [56, 58–60]. Although there are a number of potential problems with the AUC statistic [55] it is widely used as a metric for model fit since it is well understood [24], and it is the recommended metric for studies assessing the influence of life history characteristics on model performance [57]. Numerous recent studies evaluating transferability have validated the use of the AUC as an appropriate performance measure [10, 13, 61]. Although studies comparing across modeling approaches need to apply corrections to account for the different degree to which they cover the range of commission errors, this study uses a single modeling approach that calculates AUC scores on the full range of commission errors [46], therefore, AUC scores should be comparable. AUC scores for both the testing and training datasets were retained for further analyses. Clamping and multivariate environmental similarity surfaces (MESS) were also evaluated for each projected model to ensure that the climates to which data were projected were within the range of environments sampled by the training data [18]. The equal specificity and sensitivity threshold was used to create a binary presence or absence matrix for each cell. While the choice of threshold criterion is somewhat arbitrary, this particular threshold was selected because it equalizes the model’s ability to correctly predict a presence if the species is present, and to correctly predict an absence when the species is absent [53, 62].

Many studies examining the impact of life history traits have recovered a pattern whereby models for narrow ranging, endemic species have higher model accuracy than those for widespread species as assessed by AUC scores [5, 10, 63, 64]. However, for species occupying a restricted range relative to the extent of the background area modeled, AUC scores can be elevated as an artifact of modeling due to sampling prevalence and sample size [57], and pseudo-absences [14, 65, 66]. By explicitly evaluating the effect of background extent on AUC scores for these species, the artifacts that arise from modeling should become evident [66]. To untangle artifacts of modeling from effects of life history traits, I generated a second set of models for narrow ranging endemics using a reduced background extent (extent restricted to states where a CBC circle recorded a presence) and then compared the AUC scores from the two models sets using t-tests to assess if background extent impacted estimates of model performance. Since sampling size and the number of background points were held constant, and therefore neither sampling prevalence or sample size should be affecting differences among the sets of models, a significant difference between the models (full extent versus reduced extent) suggests that pseudo-absence selection influences AUC scores. If the models do not differ statistically, the higher AUC scores recovered for narrow ranged species may reflect a true effect of the underlying species ecology.

### Model Parameterization, Performance, and Transferability

AUC scores from the testing (AUC_test_) and training (AUC_train_) data sets were interpreted using the general guidelines outlined by Swets [67]: AUC < 0.9 = excellent, 0.9 > AUC > 0.8 = good, 0.8 > AUC > 0.7 = fair, 0.7 > AUC > 0.6 = poor, and 0.6 > AUC = fail. When used together AUC_train_ and AUC_test_ provide additional insight into model fit and validation [54]. For each model I performed an internal model evaluation (IE) by using the ratio of AUC_test_ and AUC_train_ (IE = AUC_test_ /AUC_train_). In this ratio, a value greater than 1, may indicate that the model is over-parameterized. I also used the difference between AUC_test_ and AUC_train_ (AUC_Diff_ = AUC_train_-AUC_test_) as a measure of model over-fit [52]. The differences in model fit and validation as measured by IE and AUC_Diff_ between interpolative and extrapolative models were assessed using paired t-tests.

To gauge model transferability I examine the magnitude of over- or under- prediction and assess the overlap between modeled ranges. This approach assumes that the Im better represents the true distribution than does the Em [16, 68]. External model evaluation was performed using two AUC -based Transferability Indices to gauge the accuracy of the extrapolative models. Both TI_H_ (TI_H_ = AUC_(Em)_ / AUC_(Im)_), developed by Heikkinen *et al*. [37] and TI_W_ (TI_W_ = (1-(AUC_Diff(Em)_)) /(1-(AUC_Diff(Im)_)) developed here, are simple ratios in which values less than one indicate that the interpolative models are providing more accurate predictions than the extrapolative models. Values close to one are indicate equivalent accuracy of interpolative and extrapolative models. The second index, TI_W_ differs from TI_H_ by incorporating the internal evaluation into the ratio. I also use metrics to quantify mismatch between the interpolative models (Im) and the extrapolative models (Em). The metrics used here are from [69] and include the relative range size (RRS) and the overlap index (OI). They are intended to provide insight into the performance of the transferred extrapolative models (Em) relative to the interpolative models (Im). The relative range size (RRS) (if Im < Em RRS = Em/Im -1, else RRS = -1(Im/Em-1) is a measure of the over- or under- prediction of suitable area of the Em relative to the Im. A positive number means that the Em over-predicts the range, a negative value means it under-predicts the range, and a value close to 0 indicates model similarity [16]. The Overlap Index (OI) (OI = (O/Im)) quantifies agreement between the predicted and projected species range. OI records the amount of congruence between the Im and Em models. It is a simple ratio that quantifies the overlap between the Im and Em models with respect to the Im. Values range from zero to one. A value close to one indicates that the Em performs well in predicting the species range. A low value indicates little agreement between the models.

### Life History Characteristics, Model Accuracy, and Transferability

The next step in these analyses is to relate differences in model accuracy and transferability to differences in life history strategies among species. Specifically addressing (a) which life history characteristics influence model performance and (b) which life history traits influence model transferability. Contingency tables were used to examine interactions among life history characteristics (migratory status, range status, habitat type, conservation status, population trend, and body size). The relationship between life history characteristics and ENMs was assessed using generalized linear models (GLMs). Homogeneity among residuals and other assumptions for application of GLMs were checked using graphical methods following guidance from Zuur *et al*. [70]. GLMs were implemented using the Gaussian family with the identity link. Five variables that measure model accuracy (AUC_avg_) and transferability (RSS, OI, TI_H_, TI_W_) were treated in turn as the response variable and modeled using the *drop1* function in R [71] which gives the significance of each variable after all remaining variables are controlled for. Predictor variables included body mass, range (wide or narrow), range (common or rare), migration status, habitat, conservation status, and population trend. Significance was assessed using analysis of deviance with the Chi Square distribution, and the best-fit model was determined using the likelihood ratio test of the AIC scores. I then used the non-parametric Wilcoxon test to evaluate both model accuracy and transferability as they relate to range components, and the Kruskal-Wallis test to examine differences among model performance as measured by the mismatch statistics and with respect to each of the life-history and range characteristics.

## Results

### Model Performance and Transferability

Thirty-seven North American bird species were evaluated using the CBC data (Table 1). Clamping was non-existent in these analyses, and there was very little dissimilarity of multivariate environments. VIF scores indicate that there is some collinearity among the predictor variables (Table 2). For all five decades we found consistent evidence for collinearity for annual mean temperature (bio 1), min temperature of the coldest month (bio 6), precipitation of the wettest quarter (bio 16), and precipitation of the driest quarter (bio 17). Three additional variables were also identified as collinear during different decades: max temperature of warmest the month (bio 5), mean temperature of the warmest quarter (bio 10), and annual precipitation (bio 12) (Table 2). Over the short time examined here, the relationships among variables were not constant.

**Table 2:** Collinear environmental predictor variables for each decade detected by VIF analyses with a threshold of 0.9.

Decade	Collinear Environmental Predictors (>0.90)
1950s	1, 5, 6, 16, 17
1960s	1, 6, 12, 16, 17
1970s	1, 6, 10, 16, 17
1980s	1, 6, 10, 12, 16, 17
1990s	1, 6, 10, 16, 17

Variable Key: 1 = annual mean temperature, 6 = min temperature of the coldest month, 16 = precipitation of the wettest quarter, 17 = precipitation of the driest quarter, 5 = max temperature of warmest the month, 10 = mean temperature of the warmest quarter, and 12 = annual precipitation.

Both testing and training AUC values had high discriminatory power for Im were excellent for 255 models, good for 72 models, fair for 35 models, poor for 8, and failed for 0. For the Em AUC values were excellent for 511 models, good for 156 models, fair for 60 models, poor for 10, and failed for 3. When averaged across decades AUC_test_ values were excellent for 22, good for eight, and fair for seven species. For AUC_train_ values were excellent for 28, good for eight, and fair for one species. The internal and external validations suggest that the Im and Em models are similarly parameterized and well calibrated (S2 Table). Both of the transferability indices generally provide similar results and suggest that the models are highly transferable (S2 Table). The RRS statistic found that many models had values close to 0 indicating strong agreement between the Im and Em, however there were a number of models that suffered from over- and under-prediction (S2 Table). While under-prediction was more common than over-prediction, the magnitude of over-prediction was larger (S2 Table). The OI statistic revealed that for many models there was high-predicted overlap between the Im and Em models, although there were some exceptions (S2 Table). While most of the metrics suggest that the models are accurate, well fit, and transferable, there are some Em that either over or under-predicted the Im, as well as some Em that had low overlap with the Im, suggesting that these discrepancies arise either as an artifact of modeling, or that some other factor potentially related to life history characteristics affects model prediction and transferability.

### Life History Characteristics

Of the species included in these analyses, eleven had stable population trends while twenty-six had unstable population trends (eight increasing and eighteen decreasing), nine were residents, twelve were Neotropical migrants, and sixteen were temperate migrants (Table 1). Range status and migratory status were not independent, with temperate migrants mainly falling within the WC range status group (Chi-square test yielded a p-value of 0.036). Habitat preference, body size, and conservation status were all independent. Maps from ebird suggest that all but 5 species (Sage Grouse, Prairie Chicken, Yellow-billed Magpie, Saltmarsh Sparrow, Mountain Plover) have access to and disperse through a large proportion of the extent of the area modeled in this study.

Model accuracy was significantly affected by several life history characteristics (Table 3). Range, migration and habitat were important predictors of model accuracy.

**Table 3:** Generalized Linear Models indicating the predictors for each measure of model performance.

Test Variable	Life History Predictor Variable	SS	RSS	AIC	F-value	*P*
AUC (avg)			0.040868	-223.91		
	Body Mass	0.001130	0.041998	-224.90	0.6360	0.4333
	**Range—Wide/Narrow**	**0.012498**	**0.053366**	**-216.04**	**7.0338**	**0.0142**
	Range—Common/Rare	0.006682	0.047549	-220.31	3.7605	0.0648
	**Migration**	**0.045930**	**0.086798**	**-200.04**	**12.9247**	**0.0002**
	**Habitat**	**0.024105**	**0.064973**	**-216.75**	**2.7133**	**0.0455**
	Conservation Status	0.003800	0.044668	-222.62	2.1386	0.1572
	Population Trend	0.004907	0.045775	-223.71	1.3808	0.2714
TI_H_ (avg)			0.023153	-244.93		
	Body Mass	0.000171	0.023324	-246.66	0.1702	0.6837
	Range—Wide/Narrow	0.001708	0.024861	244.30	1.6970	0.2056
	Range—Common/Rare	0.000144	0.023296	-246.70	0.1425	0.7093
	Migration	0.001057	0.024209	247.28	0.5249	0.5985
	Habitat	0.002759	0.025912	-250.77	0.5482	0.7380
	Conservation Status	0.000098	0.023251	-246.78	0.0974	0.7578
	Population Trend	0.001194	0.024347	-247.07	0.5930	0.5609
TI_W_ (avg)			0.016132	-258.30		
	Body Mass	0.000045	0.016177	-260.20	0.0634	0.8034
	Range—Wide/Narrow	0.001059	0.017191	-257.95	1.5095	0.2316
	Range—Common/Rare	0.000076	0.016208	-260.13	0.1084	0.7450
	Migration	0.000901	0.017033	-260.29	0.6423	0.5353
	Habitat	0.001537	0.017669	-264.93	0.4382	0.8172
	Conservation Status	0.000003	0.016135	-260.29	0.0043	0.9484
	Population Trend	0.000605	0.016737	-260.94	0.4312	0.6549
RRS			2753.7	187.46		
	Body Mass	1.921	2755.6	185.49	0.0160	0.9003
	Range—Wide/Narrow	24.953	2778.7	185.80	0.2084	0.6523
	Range—Common/Rare	17.050	2770.8	185.69	0.1424	0.7094
	Migration	171.608	2925.3	185.70	0.7167	0.4990
	Habitat	150.561	2904.3	179.43	0.2515	0.9347
	Conservation Status	159.642	2913.3	187.55	1.3334	0.2601
	Population Trend	121.690	2875.4	185.06	0.5082	0.6082
OI			0.44631	-135.45		
	Body Mass	0.000000	0.44631	-137.45	0.0000	0.9987
	Range—Wide/Narrow	0.001405	0.44771	-137.34	0.0724	0.7903
	**Range—Common/Rare**	**0.103077**	**0.54938**	**-129.76**	**5.3120**	**0.0306**
	Migration	0.029849	0.47615	-137.06	0.7691	0.4750
	Habitat	0.033594	0.47990	-142.77	0.3462	0.8794
	Conservation Status	0.002947	0.44925	-137.21	0.1519	0.7003
	Population Trend	0.093748	0.54005	-132.40	2.4156	0.1116

Significant predictors (p<0.05) are in bold.

**Fig 2:**
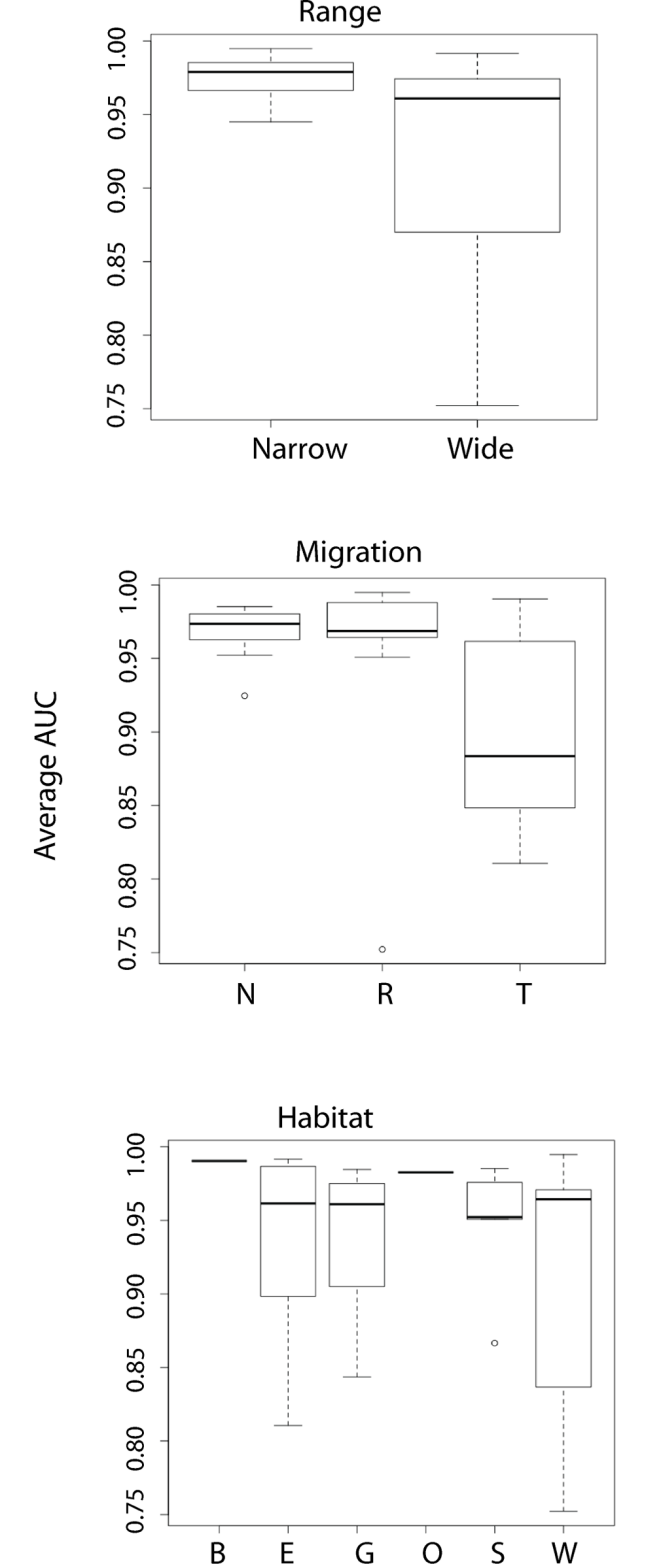
Boxplots of differences in model accuracy (measured by AUC) for species grouped by range (WC: widespread common; WR: widespread rare; NEC: narrow endemic common; NER: narrow endemic rare), migration (N: Neotropical; R: resident; T: temperate), and habitat (S: scrub, W: woodland, G: grassland, E: wetland, D: desert, B: shoreline.

For range, widespread versus narrow species distributions but not the commonness or rarity of the species impacted the model accuracy, with narrowly distributed species having higher model accuracy than widespread species (Fig 2). To determine if this finding reflects an artifact of modeling or an effect of life history trait, AUC scores for models for narrowly distributed species were re-evaluated with a reduced background extent and then compared to models with the full background extent. The two sets of models were found to be statistically significantly different (AUC_test_: t = 5.4379, p-value = 0.000) which suggests that these differences arose as an artifact of modeling.

Models for both Neotropical migrants and residents had high levels of model accuracy, while models for temperate migrants had markedly reduced accuracy (Fig 2). Among the six major habitat categories included in these analyses, model accuracy for wetland species exhibited a large amount of variation, although the mean AUC was high (Fig 2). The Wilcoxon test found support for significant differences in accuracy for both range components (e.g. widespread vs narrow, common vs rare) (Table 4).

**Table 4:** Wilcoxon tests evaluating differences in model accuracy and transferability indices with regard to range characteristics.

	AUC	TI_H_	TI_W_
Narrow or Widespread	187.5 **[0.038]**	51 **[0.007]**	62 **[0.023]**
Common or Rare	92 **[0.021]**	228 [0.068]	222 [0.101]

Significant p-values <0.05 are in bold.

**Fig 3:**
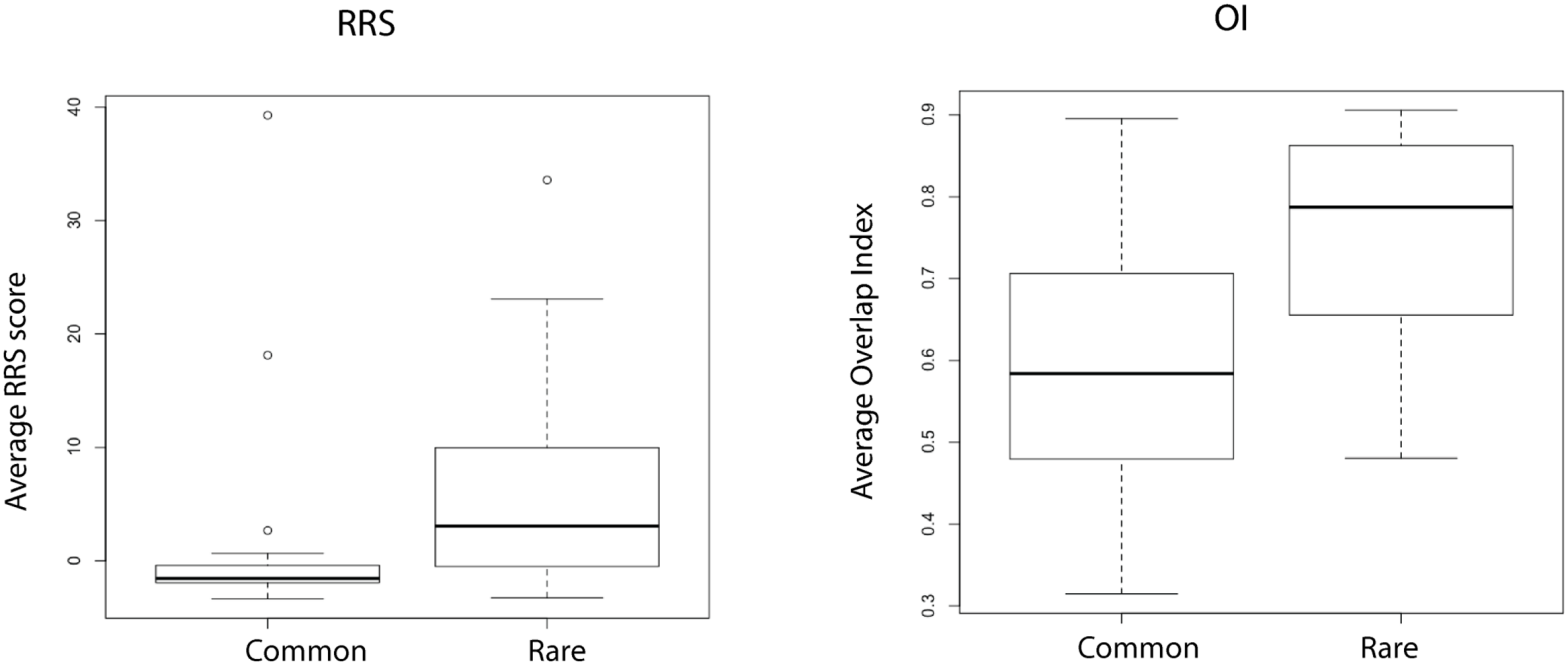
Boxplots depicting differences in average relative range size (RRS) and average overlap index (OI) between common versus rare species.

The commonness or rarity of the species (range) was a significant predictor for OI but not RSS (Table 3, Fig 3). The OI was higher for rare birds indicating better agreement between the Im and Em models than for common birds, Interestingly, GLMs did not find any of the life history characteristics examined here to be significant predictors of model transferability as measured by TI_H_ or TI_W_ metrics (Table 3), although the Wilcoxon test did recover significant support for differences in transferability for narrow versus widespread species (Table 4).

**Fig 4:**
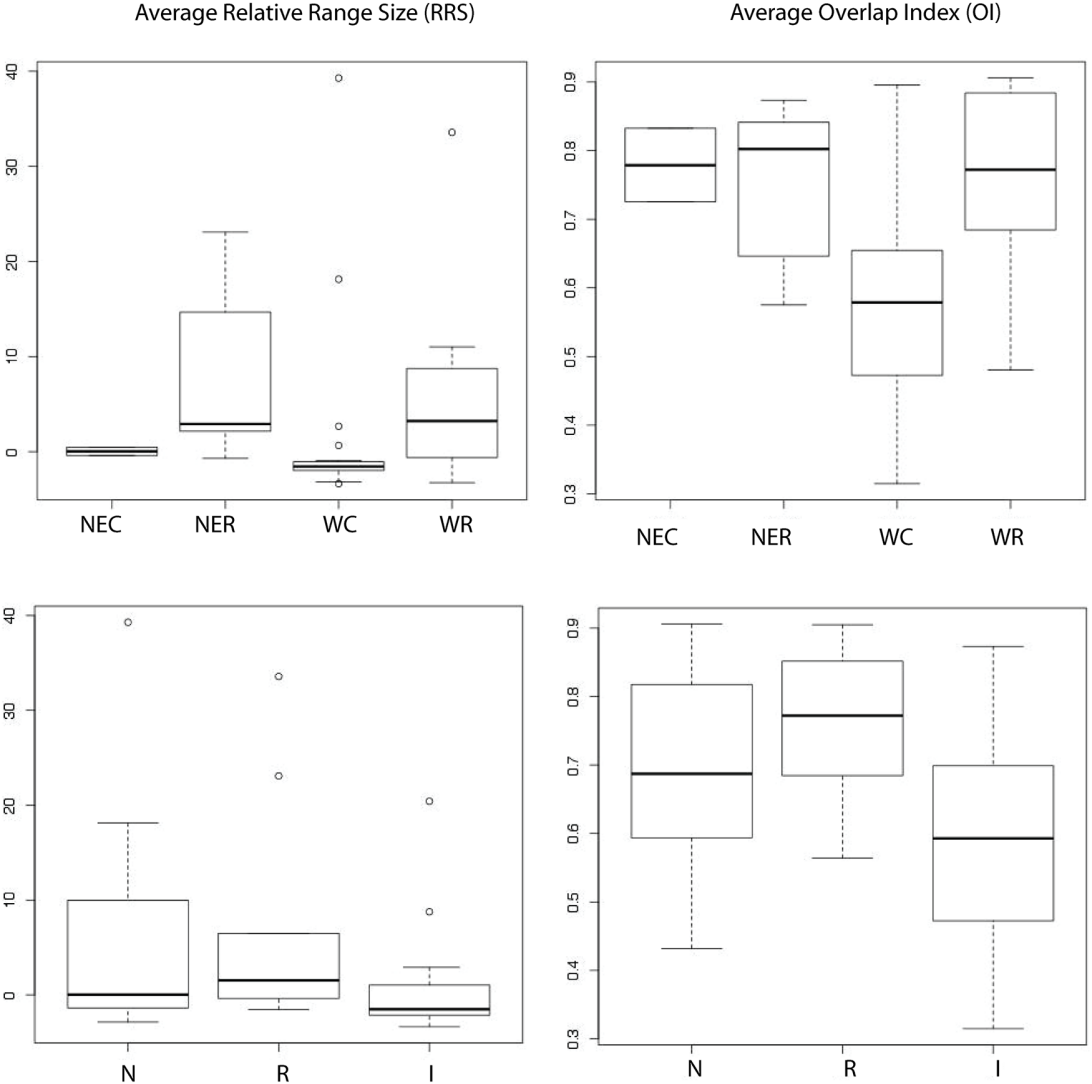
Boxplots of species grouped by range status (top row) (WC: widespread common; WR: widespread rare; NEC: narrow endemic common; NER: narrow endemic rare) and migratory status (bottom row) (N: neotropical; R: resident; T: temperate). Relative Range Size (RRS) values close to 0 indicate good agreement, positive values indicate over-prediction and negative values indicate under-prediction, Overlap Index (OI) values range from 0 (no overlap) to 1 (full overlap).

Among group comparisons of means by range, migratory status, and IUCN population status revealed statistically significant differences in mean RRS (Table 5). NEC, NER, WC, and WR categories had widely differing group means, models for rare species tended to over predict range, while models for common species did a better job of capturing range dynamics. The mean RRS values for temperate migrants was 0.69 ± 6.09 sd suggesting that the Im and Em predicted similar ranges for these birds, while for Neotropical migrants and residents the mean RRS values were 5.956 ± 12.33 sd and 7.332 ± 12.42 sd respectively, suggesting a tendency towards over-prediction. As expected, for species with an increasing population trend, the mean RRS value was negative (-1.623 ± 1.233 sd) indicating a tendency towards under prediction, while for stable species and species with a decreasing population status, the mean RRS values were positive (5.786 ± 10.97 sd and 5.435 ± 11.49 sd respectively), indicating a tendency towards over prediction. When evaluating OI with respect to the four range scenarios, the average OI value for widespread common species was lower than for the other range scenarios, and the means among the different range scenarios were statistically significantly different (Table 5, Fig 4). The OI also differed among migratory groups, with temperate migrants having a lower average OI score than resident or Neotropical migrants (Table 5, Fig 4). Group means among IUCN population status categories were also significantly different, with the highest OI value recorded for species with stable populations (0.74 ±0.17 sd), and the lowest for those with increasing populations (0.543 ± 0.14 sd).

**Table 5:** Results of Kruskal-Wallis tests evaluating among group differences when species are grouped by range status, migratory status, habitat type, conservation status, IUCN population status or National Audubon population status.

	Range Status	Migratory Status	Habitat	Conservation Status	IUCN	NAS
RRS	0.009**	0.031*	0.53	0.153	0.046*	0.082
OI	0.008**	0.038*	0.715	0.816	0.05*	0.091

Significance at alpha < 0.05 is demarcated by an *, and alpha < 0.01 is demarcated by **.

## Discussion

In this study I modeled serially sampled survey sites over a 50-year period in a region where climates have remained relatively steady through time. I expected that if the models were performing well then they should have high transferability, and therefore differences in transferability should then be due to other factors such as life history traits or artifacts arising during modeling. In agreement with results from McPherson and Jetz [5], differences among bird species ecologies impacted the predictive accuracy of models. In both studies narrowly distributed species were better modeled than widespread species and wetland species models were often less accurate than models for species in other habitats. However, in this study, the recovered differences between models for narrow versus widespread spread species appears to be an artifact of the effect of the selection of pseudo-absences on the AUC statistic. This occurs when using the same modeling extent for all species, since narrow range species occupy a smaller subset of the entire area relative to the modeled extent [14, 57, 65]. VanDerWal et al. [66] found that pseudo-absences drawn from too large of an area relative to the species range can lead to inflated AUC statistics, which is what was observed in this study. Many studies standardize the extent of the area modeled across species of varying range sizes, careful consideration of the potential effect of the modeled extent to the range size of the species is warranted if AUC is used as a metric for model accuracy, since individual models for narrow ranged species may not actually perform as well as their AUC scores suggest.

Converse to results from McPherson and Jetz [5], this study found that the models for temperate migrants tended to have lower accuracy than those for Neotropical migrants or resident species. Different studies have come to different conclusions regarding how well models capture the distributions of migrant versus resident birds [63, 72], suggesting that this may vary across geographies and assemblages.

The performance of ENMs built using historical climate and occurrence data varied in their ability to predict “future” species distributions. In this study models were projected to highly similar analogous environments, under the expectation that they should transfer well [61]. The testing AUC scores for the models were overall quite high, and the models were well calibrated with only a slight tendency toward over-parameterization (which is less problematic than under-parameterization [52]). Although the transferability scores were generally high, the transferability of the models varied. While the GLMs did not find any of the life history traits to be significant predictors of transferability as measured by TI_H_ or TI_W_, the OI statistic suggests that commonality versus rarity of species across their distributional ranges may play an important role in the predictive ability of extrapolated models. In this study, this was the only life history character that significantly predicted overlap. In contrast, the Wilcoxon test suggests that there are statistically significant differences in transferability for narrow versus widespread species, but not for common versus rare species.

ENMs can become erratic when transferring projections in space and time [6, 10]. While this study used internal and external validations to ensure that models were well parameterized, accurate, and transferable, some models suffered from over- or under-prediction. Over-prediction could emerge as an artifact of thresholding, or as a by-product of model parameterization. If a particular threshold was always more lenient (lower) than other thresholding approaches than ranges would always tend towards over-prediction, however this was not the case. If the models were over- parameterized then the expectation is that the transferred models would under-predict the range, while the converse is true for under-parameterized models. In some studies, MaxEnt has been known to remove too many variables, resulting in over-predicted range sizes due to under-fit models [16]. Here the high degree of over-prediction for some species suggests under-fitting, although the fit statistics indicate that the models are well parameterized.

Although it has been suggested that collinear predictors do not affect MaxEnt model building, but instead impact model interpretation [73, 74], the inclusion of variables that are collinear can lead to over-fit models and potentially to the elimination of biologically relevant variables [40]. These models are expected to under-predict the range [51], and indeed, this accords with findings from Braunisch [47] in which complex models (retaining highly collinear predictor variables) performed better than simpler models (retaining independent variables), despite potential over-fit and under-prediction. Although we did observe examples of under-prediction in this study, it was of small magnitude, suggesting that collinearity is not driving the results.

An alternative explanation is that biotic interactions may play a limiting role in determining the species distribution and may be more important than climate in determining range limits for these species. These factors can include dispersal limitation and issues of access to suitable environments, or biotic interactions such as competition, or biotic dependencies on particular vegetative structures [75]. Dispersal limitation and historical access play a significant role in shaping distributions at evolutionary scales [76]. Dispersal limitation in particular could impact modeling if the extent of the modeled area is greater than the dispersal capabilities of the species during the timeframe under evaluation [77]. While experimental evidence for extreme dispersal limitation exists for some tropical forest bird species [78], the birds in this study are temperate species (expected to have high dispersal relative to tropical species [79, 80]), and the majority of them are migratory suggesting that dispersal limitation should not affect most of the species. Frequency maps generated in ebird suggest that all of the widespread and many of the “narrow” range species are able to access habitats throughout the modeled region, thus dispersal limitation is not a major driver of the species distributions in this study. Although climate alone has been shown to do a good job predicting distributions for birds [75], biotic dependencies related to habitat have been shown to be important in structuring North American bird distributions [75, 81, 82]. This study did not take into account habitat changes through time and instead focused only on abiotic environmental factors, which could have some important implications. For example, if suitable habitat decreased or fragmented, one might expect birds that closely track that habitat, to have reduced ranges relative to predicted ranges based solely on climatic factors. It is possible that critical environmental components, such as habitat, drive the distributions of some species examined in this study, and these components are not captured in the models. This could result in under-fit models and the over-predicted distributions found for some species. This study did find that habitat was an important predictor of model performance, although, it was not a significant predictor of transferability, and there were no significant statistical associations for habitat and transferability.

Another related possibility is that species are not consistently tracking the same set of climate variables in time. A variable might be important in determining range barriers at one time, but is not as important in a different time period [83, 84]. Rubidge *et al*. [15] found that species distributions within a period could be best explained by climatic variables, but that other environmental variables played an important role in range changes between times. For species such as this, ENMs trained solely on climatic variables from one time period will likely not do an adequate job in capturing range dynamics. The inclusion of additional environmental data such as vegetation might provide better predictive ability than climate alone. Multi-species modeling approaches may also better capture predicted range changes if species interactions underlie current range limits [15].

The decreased transferability found in this study could have potentially arisen due to some of the specific characteristics of the data utilized in these analyses. For some species, the range limits of the winter range extend beyond the borders of the continental United States, and these areas were not used in constructing ENMs. Projecting to highly novel climates, can lead to decreased transferability if the entire geographic range is not sampled [85]. This arises because some combinations of climatic variables are not included during model building, which can result in models that do not capture the upper and/or lower ends of the environmental envelope [85]. This could underlie some of the instances of under-prediction found here, but does not explain over-prediction. Furthermore, the climates in these analyses are analogous across all decades. There is no evidence to suggest that novel climatic combinations existed during the 50 years (as assessed by the absence of clamping and with the MESS statistic). As such, models should be both highly accurate and temporally transferable within the continental United States.

Another potential issue is that the CBC data, like many historical data sources may contain biases. In historical databases species misidentifications, heterogeneous collecting effort and intensity, and geographical/environmental sampling biases may exist [86]. This study implemented some measures to correct for these confounding issues; (a) species observed less than 5% of survey years at a survey site were considered potential misidentifications and were removed for these analyses, (b) presence and absence at any site for each timespan was determined by ten years worth of surveys thus minimizing the effects of a low survey-effort year, (c) these data were recorded as simple presence or absences rather than as abundances, which also alleviates confounding effects from uneven survey effort, and (d) systematic sampling (*sensu* [50]) was used to correct for geographic clustering of presence points. However, any potential skew in the geographical distribution of the CBC circles, such as under sampling in a particular region has not been corrected.

Indeed, the primary underlying difference in model accuracy and transferability in this study appears to relate to species life history characteristics. Life history traits impart a strong signature on the nature of species ranges and abundances, and although they clearly impact both accuracy and transferability, they are often overlooked. This raises the question of how to best assess and integrate life history trait impacts on accuracy and transferability into modeling efforts, particularly those that use forecasting to assess conservation issues under projected future climate change. As has been argued [22], conservation efforts should consider climate change in their planning.

Studies examining life history traits and model accuracy and transferability are needed for a wider group of organisms, especially for taxa that are of high conservation concern and experiencing elevated rates of extinction, such as amphibians. Although high quality historical data are not available for many taxa in most part of the world, sources for these types of data are becoming available. Another option is to partition available data from existing sources such as GBIF [87], VertNet [88] at a finer temporal scale and project those data forward or backward to test ENM accuracy and transferability as it relates to life history characteristics. As more studies become available for more taxa from more regions, generalities should emerge that will provide a framework integrating model behavior and life history traits.

## Supporting Information

S1 Table The 19 Bioclim variables used in this study (obtained from WorldClim at 30 arc second resolution). (DOC)

S2 Table Accuracy, validation, transferability, and mismatch statistics for each species. (DOC)
