# Supplementary material for: Life History Traits and Niche Instability Impact Accuracy and Temporal Transferability for Historically Calibrated Distribution Models of North American Birds
Source: PLoS One. 2016 Mar 9;11(3):e0151024. doi: 10.1371/journal.pone.0151024 (PMC4784944; doi:10.1371/journal.pone.0151024)
Supplement: S1 Table — (DOC) [file pone.0151024.s001.doc]

| Bioclim 1 | Annual mean temperature |
| --- | --- |
| Bioclim 2 | Mean diurnal range |
| Bioclim 3 | Isothermality |
| Bioclim 4 | Temperature seasonality |
| Bioclim 5 | Max temperature of warmest month |
| Bioclim 6 | Min temperature of coldest month |
| Bioclim 7 | Temperature annual range |
| Bioclim 8 | Mean temperature of wettest quarter |
| Bioclim 9 | Mean temperature of driest quarter |
| Bioclim 10 | Mean temperature of warmest quarter |
| Bioclim 11 | Mean temperature of coldest quarter |
| Bioclim 12 | Annual precipitation |
| Bioclim 13 | Precipitation of wettest month |
| Bioclim 14 | Precipitation of driest month |
| Bioclim 15 | Precipitation seasonality |
| Bioclim 16 | Precipitation of wettest quarter |
| Bioclim 17 | Precipitation of driest quarter |
| Bioclim 18 | Precipitation of warmest quarter |
| Bioclim 19 | Precipitation of coldest quarter |
