# Supplementary material for: Life History Traits and Niche Instability Impact Accuracy and Temporal Transferability for Historically Calibrated Distribution Models of North American Birds
Source: PLoS One. 2016 Mar 9;11(3):e0151024. doi: 10.1371/journal.pone.0151024 (PMC4784944; doi:10.1371/journal.pone.0151024)
Supplement: S2 Table — (DOC) [file pone.0151024.s002.doc]

| **AOU** | **Species** | **Predicted Area (avg)** | **AUC (avg)** | **Avg IE (Im)** | **Avg IE (Em)** | **TI_H_ (avg)** | **TI_W_ (avg)** | **Avg. RRS** | **Avg. OI** |
| --- | --- | --- | --- | --- | --- | --- | --- | --- | --- |
| AOU 10 | *Gavia immer* | 1.9534E+12 | 0.9005 | 0.947 | 0.957 | 1.0105 | 1.0084 | -2.0196 | 0.4796 |
| AOU 172 | *Anas acuta* | 3.5580E+12 | 0.8256 | 1.15 | 1.18 | 1.0259 | 1.0207 | -1.6165 | 0.5739 |
| AOU 240 | *Buteo platypterus* | 1.2360E+12 | 0.9624 | 0.946 | 0.927 | 0.9790 | 0.9816 | 0.6573 | 0.7320 |
| AOU 242 | *Buteo swainsoni* | 6.0110E+11 | 0.9202 | 0.881 | 0.906 | 1.0284 | 1.0260 | 18.1253 | 0.8048 |
| AOU 251 | *Aquila chrysaetos* | 1.3626E+12 | 0.8601 | 0.952 | 0.961 | 1.0098 | 1.0094 | 8.7550 | 0.8262 |
| AOU 271 | *Falco mexicanus* | 2.5320E+12 | 0.8875 | 1.168 | 1.261 | 1.0796 | 1.0452 | -0.9589 | 0.6914 |
| AOU 295 | *Centrocercus urophasianus* | 2.7139E+11 | 0.9495 | 0.916 | 0.855 | 0.9334 | 0.9416 | 23.0808 | 0.8516 |
| AOU 302 | *Tympanuchus cupido* | 6.9551E+11 | 0.9579 | 1.333 | 1.307 | 0.9802 | 0.9868 | 1.5196 | 0.5754 |
| AOU 335 | *Rallus longirostris* | 5.9880E+11 | 0.9869 | 0.825 | 0.812 | 0.9840 | 0.9822 | -0.3866 | 0.6845 |
| AOU 360 | *Grus canadensis* | 2.6540E+12 | 0.9367 | 1.06 | 1.092 | 1.0297 | 1.0248 | -3.1758 | 0.3149 |
| AOU 376 | *Charadrius melodus* | 3.3880E+11 | 0.9906 | 0.901 | 0.897 | 0.9952 | 0.9954 | -0.7214 | 0.6477 |
| AOU 379 | *Charadrius montanus* | 7.1520E+11 | 0.9798 | 1.054 | 1.04 | 0.9869 | 1.0104 | 8.9287 | 0.8309 |
| AOU 382 | *Haematopus palliatus* | 1.8367E+11 | 0.9913 | 0.912 | 0.905 | 0.9923 | 0.9939 | 33.5708 | 0.9049 |
| AOU 412 | *Numenius americanus* | 9.1500E+11 | 0.9817 | 0.991 | 1.015 | 1.0240 | 1.0218 | -1.8406 | 0.5788 |
| AOU 691 | *Athene cunicularia* | 1.2782E+12 | 0.9668 | 0.993 | 0.982 | 0.9884 | 0.9892 | -1.1213 | 0.6632 |
| AOU 939 | *Melanerpes lewis* | 2.0508E+12 | 0.9589 | 0.991 | 0.987 | 0.9965 | 0.9967 | -3.2586 | 0.4806 |
| AOU 971 | *Picoides arcticus* | 1.1050E+12 | 0.9635 | 0.988 | 0.98 | 0.9919 | 0.9920 | 3.2235 | 0.7722 |
| AOU 1203 | *Tyrannus tyrannus* | 5.8359E+11 | 0.9841 | 0.933 | 0.934 | 1.0015 | 1.0011 | 39.2824 | 0.6029 |
| AOU 1252 | *Lanius ludovicianus* | 3.0860E+12 | 0.8287 | 0.928 | 0.969 | 1.0446 | 1.0443 | -1.4348 | 0.6119 |
| AOU 1317 | *Pica nuttalli* | 1.0239E+11 | 0.9949 | 0.996 | 0.987 | 0.9911 | 0.9912 | 0.4591 | 0.8324 |
| AOU 1341 | *Tachycineta bicolor* | 8.2304E+11 | 0.9643 | 1.29 | 1.265 | 0.9813 | 0.9875 | -3.3616 | 0.3172 |
| AOU 1361 | *Poecile hudsonicus* | 3.1662E+11 | 0.9641 | 1.05 | 1.128 | 1.0747 | 1.0484 | 6.4792 | 0.8841 |
| AOU 1370 | *Sitta carolinensis* | 1.6050E+12 | 0.7763 | 0.931 | 0.934 | 1.0035 | 1.0023 | -1.5635 | 0.5640 |
| AOU 1372 | *Sitta pusilla* | 8.7200E+11 | 0.9688 | 0.967 | 0.915 | 0.9463 | 0.9469 | -0.3948 | 0.7252 |
| AOU 1425 | *Regulus calendula* | 2.3823E+12 | 0.8235 | 0.975 | 0.993 | 1.0185 | 1.0181 | -2.2660 | 0.4256 |
| AOU 1483 | *Hylocichla mustelina* | 1.4207E+11 | 0.9842 | 1.005 | 0.991 | 0.9866 | 0.9874 | 11.0275 | 0.9057 |
| AOU 1575 | *Dendroica petechial* | 3.064E+11 | 0.9797 | 0.983 | 0.998 | 1.0148 | 1.0146 | 2.6732 | 0.8953 |
| AOU 1595 | *Dendroica palmarum* | 1.5318E+12 | 0.9596 | 1.018 | 1.023 | 1.0046 | 1.0042 | -2.8642 | 0.4322 |
| AOU 1804 | *Spizella breweri* | 1.0366E+12 | 0.9780 | 0.995 | 0.986 | 0.9909 | 0.9911 | -1.5546 | 0.6180 |
| AOU 1805 | *Spizella pusilla* | 2.2410E+12 | 0.8745 | 0.991 | 1.001 | 1.0100 | 1.0100 | -1.8829 | 0.5263 |
| AOU 1814 | *Ammodramus savannarum* | 1.3700E+12 | 0.9580 | 1.05 | 1.045 | 0.9946 | 0.9969 | -0.6227 | 0.7110 |
| AOU 1837 | *Calcarius ornatus* | 1.4566E+12 | 0.9427 | 0.962 | 0.976 | 1.0149 | 1.0144 | 2.9025 | 0.6447 |
| AOU 1880 | *Sturnella magna* | 2.2152E+12 | 0.8791 | 0.975 | 0.928 | 0.9524 | 0.9549 | -1.1730 | 0.7062 |
| AOU 1916 | *Icterus galbula* | 1.1772E+12 | 0.9679 | 1.013 | 1.065 | 1.0520 | 1.0415 | -1.2283 | 0.5840 |
| AOU 1931 | *Leucosticte atrata* | 9.0228E+11 | 0.9481 | 0.978 | 0.942 | 0.9639 | 0.9663 | 2.7855 | 0.8022 |
| AOU 1958 | *Coccothraustes vespertinus* | 2.4758E+12 | 0.8593 | 0.989 | 0.967 | 0.9772 | 0.9774 | -1.9451 | 0.4656 |
| AOU sms | *Ammodramus caudacutus* | 1.1469E+11 | 0.9859 | 1.034 | 1.014 | 0.9808 | 0.9822 | 20.4113 | 0.8734 |
